# DMP1 prevents osteocyte alterations, FGF23 elevation and left ventricular hypertrophy in mice with chronic kidney disease

**DOI:** 10.1038/s41413-019-0051-1

**Published:** 2019-04-25

**Authors:** Corey Dussold, Claire Gerber, Samantha White, Xueyan Wang, Lixin Qi, Connor Francis, Maralee Capella, Guillaume Courbon, Jingya Wang, Chaoyuan Li, Jian Q. Feng, Tamara Isakova, Myles Wolf, Valentin David, Aline Martin

**Affiliations:** 1 Division of Nephrology and Hypertension, Center for Translational Metabolism and Health, Feinberg School of Medicine, Northwestern University, Chicago, IL 60611 USA; 2 Department of Biomedical Sciences, Texas A&M College of Dentistry, Dallas, TX 75246 USA; 3 Division of Nephrology, Department of Medicine, and Duke Clinical Research Institute, Duke University School of Medicine, Durham, NC 27708 USA

**Keywords:** Pathogenesis, Calcium and phosphate metabolic disorders, Bone

## Abstract

During chronic kidney disease (CKD), alterations in bone and mineral metabolism include increased production of the hormone fibroblast growth factor 23 (FGF23) that may contribute to cardiovascular mortality. The osteocyte protein dentin matrix protein 1 (DMP1) reduces FGF23 and enhances bone mineralization, but its effects in CKD are unknown. We tested the hypothesis that DMP1 supplementation in CKD would improve bone health, prevent FGF23 elevations and minimize consequent adverse cardiovascular outcomes. We investigated DMP1 regulation and effects in wild-type (WT) mice and the Col4a3^−/−^ mouse model of CKD. Col4a3^−/−^ mice demonstrated impaired kidney function, reduced bone DMP1 expression, reduced bone mass, altered osteocyte morphology and connectivity, increased osteocyte apoptosis, increased serum FGF23, hyperphosphatemia, left ventricular hypertrophy (LVH), and reduced survival. Genetic or pharmacological supplementation of DMP1 in Col4a3^−/−^ mice prevented osteocyte apoptosis, preserved osteocyte networks, corrected bone mass, partially lowered FGF23 levels by attenuating NFAT-induced FGF23 transcription, and further increased serum phosphate. Despite impaired kidney function and worsened hyperphosphatemia, DMP1 prevented development of LVH and improved Col4a3^−/−^ survival. Our data suggest that CKD reduces DMP1 expression, whereas its restoration represents a potential therapeutic approach to lower FGF23 and improve bone and cardiac health in CKD.

## Chronic kidney disease: Bone protein protects the heart

Therapies based on a bone growth protein could prevent heart failure in chronic kidney disease (CKD) patients, say researchers in the USA. CKD often causes reduced bone mass and leads to left ventricular hypertrophy, a dangerous thickening of heart muscle related to over-production of the FGF23 hormone. In contrast, the dentin matrix protein DMP1, produced by bone cells, is known to reduce FGF23 levels and enhance bone growth. Aline Martin at Northwestern University in Chicago and co-workers increased the DMP1 levels in CKD mouse models through genetic modification and drugs, and found that this treatment restored regular bone mass, lowered FGF23 levels, reduced the occurrence of heart problems and led to longer lives. The findings suggest that therapies that restore DMP1 have the potential to improve both bone and heart health in CKD patients.

## Introduction

Chronic kidney disease (CKD), which affects over 10% of the population worldwide, causes progressive loss of kidney function and significant alterations in mineral and bone metabolism. These include loss of bone mass, increased susceptibility to fractures, and increased levels of circulating fibroblast growth factor 23 (FGF23).^1,2^ FGF23 is a phosphate- and vitamin D-regulating hormone produced and secreted by osteocytes. Although early FGF23 elevations in CKD may represent an adaptive mechanism to maintain normal serum phosphate by increasing phosphaturia and reducing calcitriol levels,^1,3,4^ FGF23 levels continue to rise exponentially during the progression of CKD^5,6^ and ultimately, become maladaptive. Indeed, elevated FGF23 in CKD is independently associated with cardiovascular disease and all-cause mortality,^7–10^ and is thought to contribute mechanistically to development of left ventricular hypertrophy (LVH), which is an important precursor of heart failure in patients with CKD.^11–15^ Novel therapeutic strategies are needed to target FGF23 elevation and bone and cardiac disease in CKD.

Dentin matrix protein 1 (DMP1) is an extracellular matrix pro-peptide that is also produced by osteocytes and is a member of the small integrin binding ligand N-linked glycoprotein family. DMP1 is cleaved into an active 57kDa C-terminal peptide, which is critical for adequate mineralization of bone and dentin, and *Fgf23* transcription in bone.^16–18^ By binding to calcium ions, DMP1 nucleates the formation of hydroxyapatite^19^ resulting in increased mineralization. DMP1 also exerts a protective role against osteocyte apoptosis, especially in the presence of high circulating phosphate levels.^20^ Finally, DMP1 is a major local suppressor of FGF23. Indeed, inactivating mutations of DMP1 result in autosomal recessive hypophosphatemic rickets (ARHR) in which primary overproduction of FGF23 by osteocytes leads to renal phosphate wasting, rickets and osteomalacia.^21–23^ In models of hereditary rickets, including DMP1 mutants, increased *Fgf23* transcription results from paracrine activation of FGFR1,^18,24^ and downstream activation of the calcium-dependent NFAT signaling pathway.^25^

Despite all that is known about the effects of DMP1 on bone mineralization and suppression of FGF23 production, few studies investigated the contribution of DMP1 to CKD-associated bone and mineral disorders and these yielded inconsistent results. One study reported increased DMP1 expression in bone biopsies from pediatric and young adult patients with CKD;^26^ however, another reported reduced DMP1 expression in adult patients undergoing dialysis.^27^ Interestingly, similar to increased FGF23, lower circulating DMP1 levels were also associated with cardiovascular events in patients undergoing peritoneal dialysis.^28^

In the present study, we tested the hypothesis that DMP1 deficiency in bone contributes to FGF23 elevation in CKD and associated adverse cardiac outcomes. We studied Col4a3^−/−^ mice that recapitulate many features of human CKD including progressive loss of kidney function, alterations of bone and mineral metabolism, elevations of circulating FGF23 levels, development of LVH in slow progressing B6 Col4a3^−/−^, and shortened lifespan.^29–32^ We demonstrate that CKD leads to significant alterations in osteocytes, including apoptosis, reduced DMP1 expression and activation of the calcium-dependent NFAT signaling that contributes to increased FGF23 transcription. Using genetic and pharmacologic approaches to increase DMP1 concentrations in bone of WT and Col4a3^−/−^ mice with CKD, we also show that restoration of DMP1 in bone prevents CKD-associated bone disease by reducing osteocyte apoptosis, lowers FGF23 production via an NFAT signaling pathway, attenuates LVH, and prolongs survival despite unchanged severity of kidney disease and worsened hyperphosphatemia.

## Results

### DMP1 expression is reduced in Col4a3^−/−^ mice with advanced CKD

**Fig. 1:**
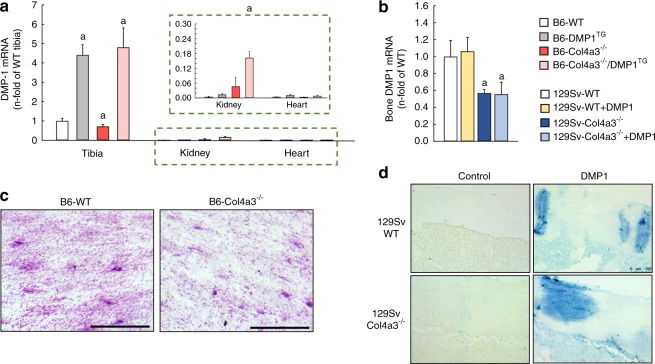
DMP1 deficiency and supplementation in Col4a3^−/−^ mice with advanced CKD. **a**–**b** DMP1 mRNA expression in whole bone, kidney and heart of **a** 20 week-old B6 WT, DMP1^TG^, Col4a3^−/−^, and Col4a3^−/−^/DMP1^TG^ mice and whole bone of **b** 9-week-old 129sv WT and Col4a3^−/−^ mice treated with mouse recombinant his-tagged DMP1 or saline control once a day for one week. **c** Bright-field microscopy of DMP1 immunostaining in cortical bone of B6 WT and Col4a3^−/−^ mice (scale bar = 50 µm). **d** His-tag specific immunodetection of DMP1 in bone of 129 Sv WT and Col4a3^−/−^ mice injected with mouse recombinant his-tagged DMP1 or saline control once a day for one week. Staining is detected in DMP1-injected animals only and shows incorporation of DMP1 around blood vessels of cortical bone and in bone marrow. Values are expressed as mean ± SEM; *n* = 5 mice/group. ^*a*^*P* < 0.05 *vs.* WT

DMP1 is mostly expressed in bone, while soft tissues such as heart and kidney express significantly lower amounts of DMP1 (Fig. 1a). Compared to age-matched wild-type (WT) mice, bone DMP1 mRNA expression was significantly reduced by 30%–40% in slow progressing B6 Col4a3^−/−^ mice (Fig. 1a) and fast progressing 129Sv-Col4a3^−/−^ mice (Fig. 1b) with advanced CKD, at 20 weeks and 9 weeks of age, respectively. Consistently, DMP1 protein expression was significantly reduced in bones from 20-week-old B6 Col4a3^−/−^ mice with advanced CKD (Fig. 1c).

We assessed the contribution of reduced DMP1 expression to CKD-associated complications using two complementary approaches. First, we increased DMP1 concentration by overexpression of the cleaved bioactive C-terminal DMP1 fragment in bone over the lifetime of B6 and 129 Sv WT (DMP1^TG^) and Col4a3^−/−^ mice (Col4a3^−/−^/DMP1^TG^). Second, we assessed the therapeutic potential of exogenous DMP1 by administering recombinant DMP1 i.p. once daily for 7 days to 129 Sv WT and Col4a3^−/−^ mice beginning at 5 and 8 weeks of age. We confirmed overexpression of DMP1 mRNA in bone of B6 DMP1^TG^ and Col4a3^−/−^/DMP1^TG^ mice compared to WT controls (Fig. 1a), and verified that recombinant DMP1 was delivered to the bones after treatment (Fig. 1d).

### DMP1 prevents alterations in bone formation and mineralization in Col4a3^−/−^ mice

**Fig. 2:**
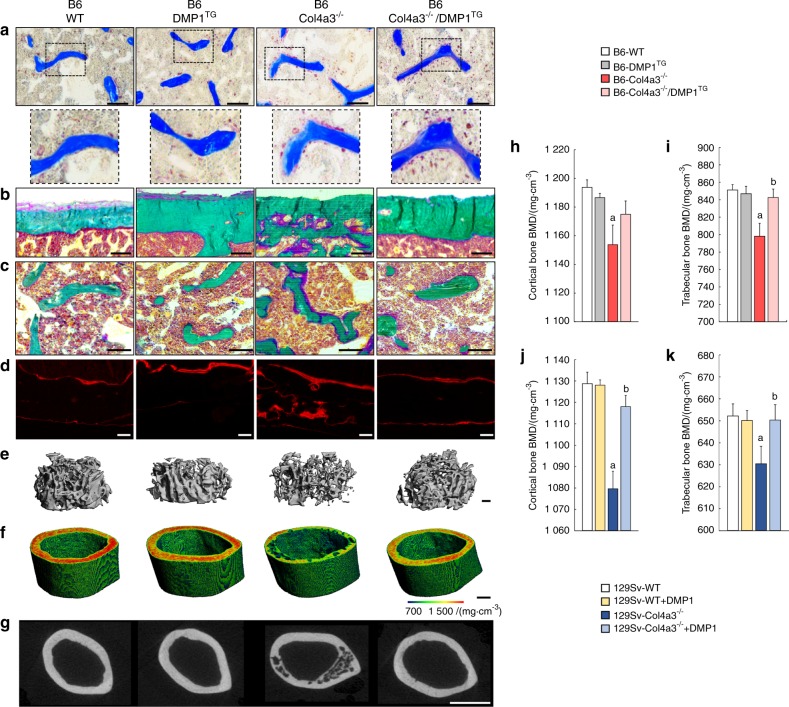
DMP1 restores bone mineralization in Col4a3^−/−^ mice with advanced CKD. Bone phenotype analyses of femurs from 23-week-old B6 WT, DMP1^TG^, and Col4a3^−/−^ and Col4a3^−/−^/DMP1^TG^ mice (**a**–**i**) and 9-week-old 129sv WT and Col4a3^−/−^ mice treated with DMP1 or saline control for 1 week (**j**–**k**). **a** Bright-field microscopy of TRAcP staining showing osteoclasts in trabecular bone (scale bar = 100 µm). **b**, **c** Bright-field microscopy of modified trichrome Goldner staining of cortical and trabecular bone (scale bar = 100 µm). **d** Fluorescent microscopy of alizarin red S double labeling in cortical bone (scale bar = 100 µm). **e**, **f** 3D microtomography of secondary spongiosa trabecular bone and midshaft cortical bone (scale bar = 200 µm). The degree of mineralization is represented by the heatmap. **g** 2D microtomography of midshaft cortical bone (scale bar = 1 mm). **h**–**k** 3D-microtomography analysis of cortical and trabecular bone mineral density (BMD) showing impaired mineralization in Col4a3^−/−^ mice and correction of impaired mineralization in Col4a3^−/−^ mice with DMP1 supplementation. Values are expressed as mean ± SEM; *n* ≥ 6/group*.* *P* < 0.05 vs. ^a^ WT, ^b^ Col4a3^−/−^

We analyzed the bone phenotype by histomorphometry and 3D microtomography. As previously described,^18^ B6 DMP1^TG^ mice have a similar bone phenotype to WT mice (Fig. 2a–g). Similar to previous reports in 129 Sv Col4a3^−/−^ mice,^31^ B6 Col4a3^−/−^ mice with advanced CKD showed a high bone turnover phenotype, characterized by significantly increased bone resorption and increased bone formation (Fig. 2a–c). In addition, B6 Col4a3^−/−^ mice displayed a significant increase in cortical bone porosity, reduced bone mineral density and diffuse alizarin red S double labeling of the bone, which indicates impaired bone mineralization (Fig. 2b–k). Consistently, bone mRNA expressions of osteoblast markers (Runx2, Sp7, Bglap, and Phex) were all reduced in B6 Col4a3^−/−^ compared to WT controls (Figure S1a), and primary osteoblasts isolated from B6 Col4a3^−/−^ bones also showed reduced mineralization in vitro compared to WT osteoblasts (Figure S1,b-c), suggesting intrinsic alterations of osteoblast differentiation in CKD. Consistent with previously described DMP1 effects on bone mineralization,^16,17,21^ bone overexpression of DMP1 reduced mineralization defects in vivo and in vitro (Figure 2b–i, S1,b-c), corrected cortical bone porosity (Fig. 2b–i), but did not attenuate increased bone resorption in B6 Col4a3^−/−^/DMP1^TG^ mice (Fig. 2a). One week of DMP1 injections to 129 Sv Col4a3^−/−^ mice with advanced CKD (Fig. 2j–k) or treatment of 129 Sv Col4a3^−/−^ primary osteoblasts with DMP1 in vitro (data not shown) led to similar results, including full correction of the bone mineralization defects. Together, our data suggest that Col4a3^−/−^ mice display significantly altered bone remodeling and mineralization, and that exogenous administration or transgenic overexpression of DMP1 corrects the bone formation and mineralization defects caused by CKD.

### DMP1 prevents CKD-induced alterations of osteocyte morphology, networks and apoptosis

**Fig. 3:**
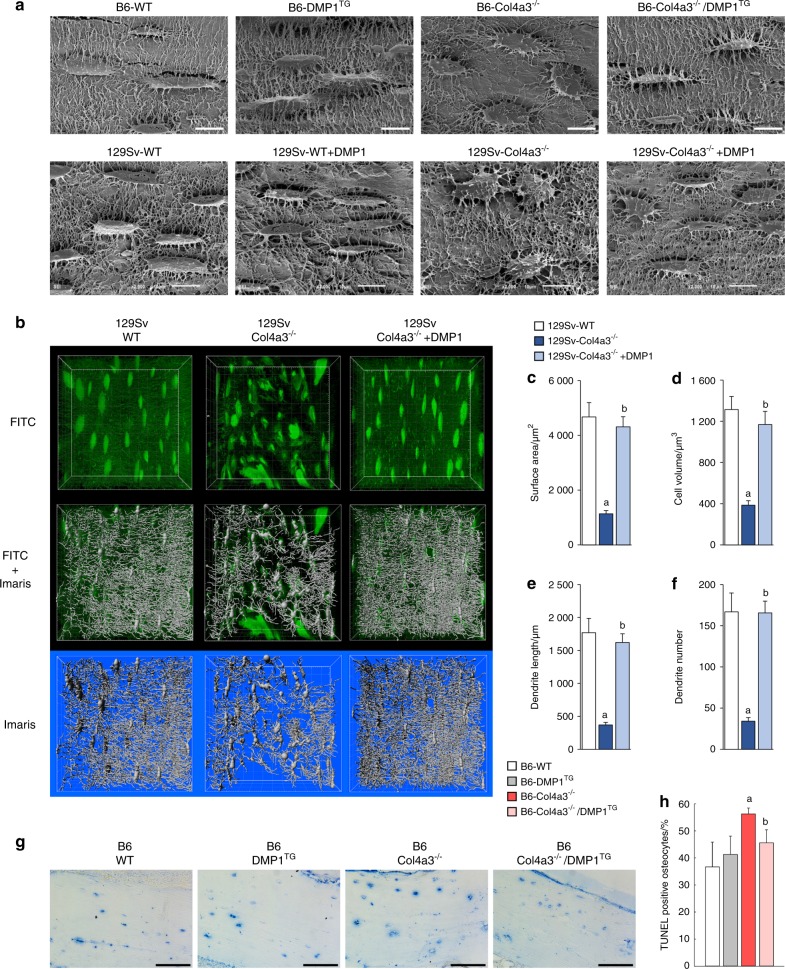
DMP1 prevents CKD-induced alterations of osteocyte morphology, networks and apoptosis. Cortical bone osteocyte analyses in 20-week-old B6 WT, DMP1^TG^, and Col4a3^−/−^ and Col4a3^−/−^/DMP1^TG^ mice and 9-week-old 129sv WT and Col4a3^−/−^ mice treated with DMP1 or saline control for one week. **a** Acid-etched scanning electron microscopy of cortical bone showing correction of osteocyte morphology in Col4a3^−/−^ bones following DMP1 supplementation (scale bar = 10 µm). **b** FITC—Imaris analysis of cortical bone showing impaired osteocyte networks in Col4a3^−/−^ cortical bone and correction of the networks following treatment with DMP1. **c**–**f** Quantification of FITC-Imaris analysis (**b**) showing impaired parameters of osteocyte networks and morphology in Col4a3^−/−^ and correction of these parameters following treatment with DMP1. **g**, **h** Bright-field microscopy of TUNEL staining on cortical bone and quantification of TUNEL-positive osteocytes. Values are expressed as mean ± SEM; *n* ≥ 3/group. *P* < 0.05 vs. ^a^ WT, ^b^ Col4a3^−/−^

Compared to normally elongated and polarized WT osteocytes, 129 Sv and B6 Col4a3^−/−^ osteocytes had a circular morphology (Fig. 3a). Osteocytes from B6 Col4a3^−/−^/DMP1^TG^ and from DMP1-injected 129 Sv Col4a3^−/−^ mice were elongated and comparable to WT osteocytes, suggesting that DMP1 fully corrected the alteration in osteocyte morphology induced by CKD (Fig. 3a). Consistent with impaired osteocyte morphology in Col4a3^−/−^ mice, FITC staining of cortical bone showed a significant decrease in osteocyte network connectivity. Using Imaris analysis,^33^ we found decreases in network surface area, cell volume, dendrite length and dendrite number in 129 Sv Col4a3^−/−^ mice (Fig. 3b–f). Osteocyte network connectivity was fully restored to normal in DMP1-injected 129 Sv Col4a3^−/−^ mice (Fig. 3b–f).

Given the altered osteocyte morphology and connectivity in CKD and the profound effects of DMP1, we hypothesized that DMP1 impacts osteocyte survival. TUNEL staining of cortical bone osteocytes was similar between B6 WT and DMP1^TG^ mice (Fig. 3g–h). However, B6 Col4a3^−/−^ displayed a significant increase in TUNEL-positive osteocytes, indicating increased apoptosis in CKD mice. CKD-induced osteocyte apoptosis was partially rescued by overexpression of DMP1 in B6 Col4a3^−/−^ mice (Fig. 3g–h), consistent with previous findings that DMP1 has anti-apoptotic effects.^20^ We further tested the effects of DMP1 in DMP1-overexpressing and control MC3T3-E1 osteoblasts cultures that we treated with increasing concentrations of the pro-inflammatory cytokine TNFα, which is known to induce osteoblast apoptosis.^34^ In support of anti-apoptotic effects of DMP1, cells overexpressing DMP1 cells were protected against TNFα-induced apoptosis (Figure S2a-b). A similar effect was observed when apoptosis was induced by hydrogen peroxide, which is another mediator of osteoblast and osteocyte apoptosis^35^ (Figure S2,c-d).

### DMP1 reduces serum FGF23 in CKD independently of kidney function

**Fig. 4:**
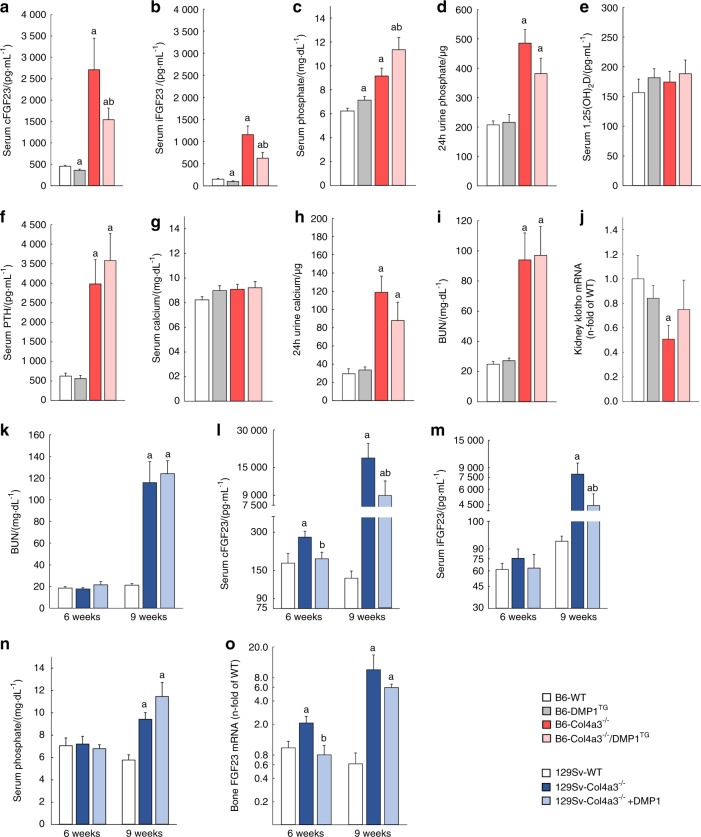
DMP1 reduces serum FGF23 in CKD independently of kidney function. Serum and urine biochemistry analysis of **a**–**j** 20 week-old B6 WT, DMP1^TG^, Col4a3^−/−^, and Col4a3^−/−^/DMP1^TG^ mice and of **k**–**n**) 6- and 9-week-old 129 Sv WT and Col4a3^−/−^ treated with mouse recombinant DMP1 or saline for one week. **a**–**d** Serum levels of total FGF23 (cFGF23), intact FGF23 (iFGF23), phosphate and 24 h urine phosphate levels. **e**–**h** Serum levels of 1,25(OH)_2_D, parathyroid hormone (PTH), calcium and 24 h urine calcium levels. **i** Serum blood urea nitrogen (BUN) levels. **j** Klotho mRNA expression in the kidney. **k** Serum BUN levels. **l–n** Serum cFGF23, iFGF23, and phosphate levels. **o** FGF23 mRNA expression in whole bone from 129 Sv WT and Col4a3^−/−^ mice with early CKD (6 weeks) and advanced CKD (9 weeks) injected daily with mouse recombinant DMP1 or saline control for one week. Values are expressed as mean ± SEM; *n* ≥ 5 mice/group. *P* < 0.05 vs. ^a^ WT, ^b^ Col4a3^−/−^

Consistent with established functions of DMP1 on FGF23 production, B6 DMP1^TG^ mice showed a 20% reduction in total serum FGF23 (cFGF23, as measured by the C-terminal FGF23 assay) and intact, biologically active, FGF23 (iFGF23, as measured by the intact FGF23 assay). This resulted in mildly increased serum phosphate and 1,25(OH)_2_D levels compared to B6 WT mice and confirms reduced end-organ effects of FGF23 in the kidney. Serum PTH and calcium levels were similar in B6 WT and DMP1^TG^ mice (Fig. 4a–h). As we recently showed,^29^ B6 Col4a3^−/−^ mice with advanced CKD expectedly showed reduced renal Klotho expression and significant alterations of mineral metabolism, including elevated serum cFGF23, iFGF23, PTH, phosphate, and calcium levels, normal 1,25(OH)_2_D levels, and increased phosphate and calcium excretion compared to B6 WT mice (Fig. 4a–j). Overexpression of DMP1 in bone of B6 Col4a3^−/−^ mice partially reduced serum cFGF23 and iFGF23, which led to further elevations in serum phosphate compared to B6 Col4a3^−/−^ mice (Fig. 4a–d). In contrast, DMP1 overexpression did not affect serum levels of PTH, 1,25(OH)_2_D or calcium in the B6 Col4a3^−/−^/DMP1^TG^ versus the B6 Col4a3^−/−^ mice (Fig. 4e–h). As expected, overexpression of DMP1 in bone had no effect on kidney function; however, renal Klotho deficiency was partially corrected in B6 Col4a3^−/−^/DMP1^TG^ mice compared to B6 Col4a3^−/−^ mice (Fig. 4i–j, Figure S3), likely due to reduced FGF23.^36^

We obtained similar results by overexpressing DMP1 in 129 Sv WT and Col4a3^−/−^ mice with fast CKD progression (Figure S4), therefore we used 129 Sv mice for repeated injections of DMP1. Daily injections of DMP1 for one week in fast progressing 6-week-old 129 Sv Col4a3^−/−^ mice with early CKD did not impact kidney function (Fig. 4k), but prevented the early significant rise in cFGF23 and the non-significant rise in iFGF23 (Fig. 4l–m). Daily injections of DMP1 for one week in terminally ill 9-week-old 129 Sv Col4a3^−/−^ mice also did not impact kidney function (Fig. 4k), but partially prevented the exponential increases in both cFGF23 and iFGF23 observed in 129 Sv Col4a3^−/−^ control mice and consequently resulted in further increased serum phosphate levels (Fig. 4l–n). The pattern of bone *Fgf23* mRNA expression paralleled circulating FGF23 levels. While one week of DMP1 injections was sufficient to completely prevent the early increase in *Fgf23* mRNA expression in 6-week-old 129 Sv Col4a3^−/−^, the same treatment only partially corrected *Fgf23* mRNA expression in 9-week-old 129 Sv mice with advanced CKD (Fig. 4o). In 9-week-old DMP1-injected 129 Sv Col4a3^−/−^ mice, serum 1,25(OH)_2_D levels remained low despite the partial correction of FGF23 levels, which resulted in further elevations of PTH (Figure S5). In aggregate, our results demonstrate that DMP1 decreases FGF23 levels in CKD, independently of mode of administration, age, mouse strain, kidney disease progression, phosphate, or PTH levels.

### DMP1 inhibits *Fgf23* transcription through regulation of NFAT1 signaling

**Fig. 5:**
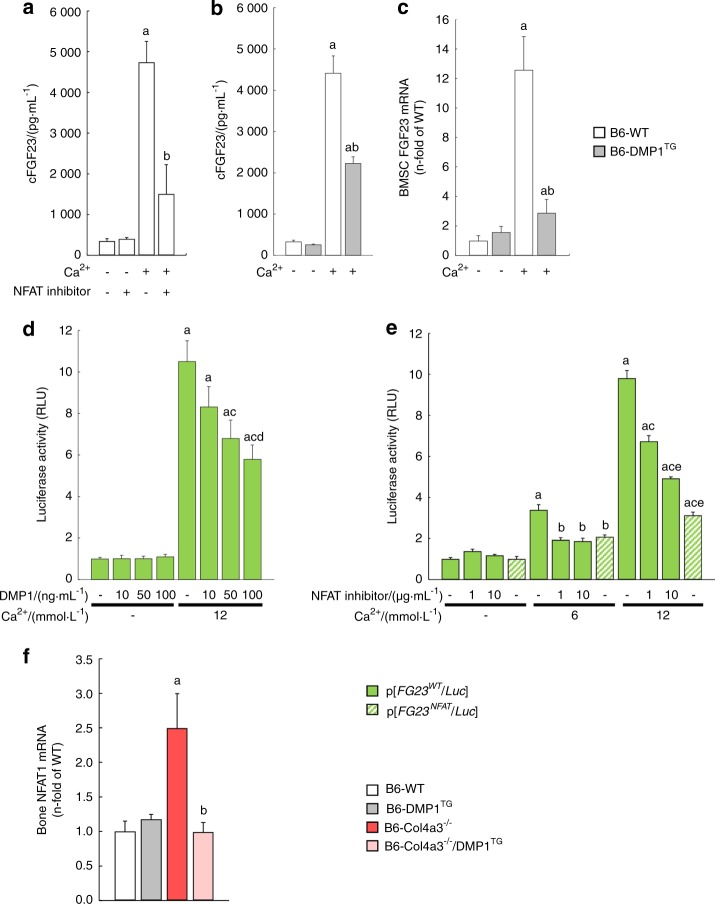
DMP1 inhibits FGF23 transcription through regulation of NFAT1 signaling. **a**, **b** Serum levels of total FGF23 (cFGF23) in B6 WT and DMP1^TG^ mice 6 h after saline, calcium chloride (3%) and/or NFAT inhibitor (10 µg·g^–1^) treatment. **c** FGF23 mRNA expression in untreated and calcium-treated (12 mmol·L^–1^) primary osteoblasts (BMSCs) isolated from B6 WT and DMP1^TG^ mice. **d**, **e** FGF23 promoter activity in MC3T3-E1 osteoblasts transfected with an intact (*p[FGF23*^*WT*^*/Luc]*) or NFAT mutant (*p[FGF23*^*NFAT*^*/Luc]*) FGF23 promoter reporter and treated with mouse recombinant DMP1, calcium and/or NFAT inhibitor. **f** NFAT1 mRNA expression in cortical bone of B6 WT, DMP1^TG^, Col4a3^−/−^, and Col4a3^−/−^/DMP1^TG^ mice. Values are expressed as mean ± SEM; *n* ≥ 5/group. *P* < 0.05 vs. ^a^ WT or control, ^b^ Col4a3^−/−^ or calcium (3% and 6 mmol·L^–1^), ^c^ calcium (12 mmol·L^–1^), ^d^ calcium (12 mmol·L^–1^) + DMP1 (10 ng·mL^–1^), ^e^ calcium (12 mmol·L^–1^) + NFAT inhibitor (1 µg·mL^–1^)

Calcium is a potent stimulator of *Fgf23* transcription^37^ and the *Fgf23* promoter contains a putative NFAT response element.^25,38^ Consistently, circulating FGF23 levels dramatically increased 6 h after a single injection of calcium in B6 WT mice, and co-treatment with the NFAT inhibitor, 11R-VIVIT, partially prevented this increase (Fig. 5a). Similar to NFAT inhibition, DMP1 overexpression partially inhibited the increase in serum FGF23 levels in calcium-injected B6 DMP1^TG^ mice compared to calcium-injected B6 WT mice (Fig. 5b). In addition, *Fgf23* mRNA expression increased by 12-fold in calcium-treated primary osteoblasts isolated from B6 WT mice, but only by three-fold in B6 DMP1^TG^ primary osteoblasts, suggesting that calcium and DMP1 converge on a common pathway to regulate *Fgf23* transcription (Fig. 5c). Therefore, we tested the hypothesis that DMP1 inhibits *Fgf23* transcription through inhibition of the calcium-NFAT pathway in osteoblast cells. Indeed, calcium treatment induced a dose-dependent activation of *Fgf23* promoter activity in MC3T3-E1 osteoblasts expressing an *Fgf23* promoter reporter (*p[FGF23*^*WT*^*/Luc]*),^39^ which was prevented either by co-treatment with increasing doses of DMP1 (Fig. 5d), increasing doses of NFAT inhibitor or by mutation of the NFAT response element of the *Fgf23* promoter (*p[FGF23*^*NFAT*^*/Luc]*) (Fig. 5e). These data suggest that calcium stimulates *Fgf23* transcription through NFAT activation and that DMP1 blocks calcium-induced *Fgf23* transcription in osteoblasts. In slow progressing B6 Col4a3^−/−^ mice, bone *Nfat1* mRNA expression was significantly increased, whereas lifelong DMP1 overexpression in B6 Col4a3^−/−^/DMP1^TG^ mice prevented this increase (Fig. 5f), suggesting that lack of DMP1-mediated suppression of NFAT-induced *Fgf23* transcription contributes to FGF23 excess in CKD.

### DMP1 prevents LVH and prolongs lifespan independently of blood pressure and CKD severity

**Fig. 6:**
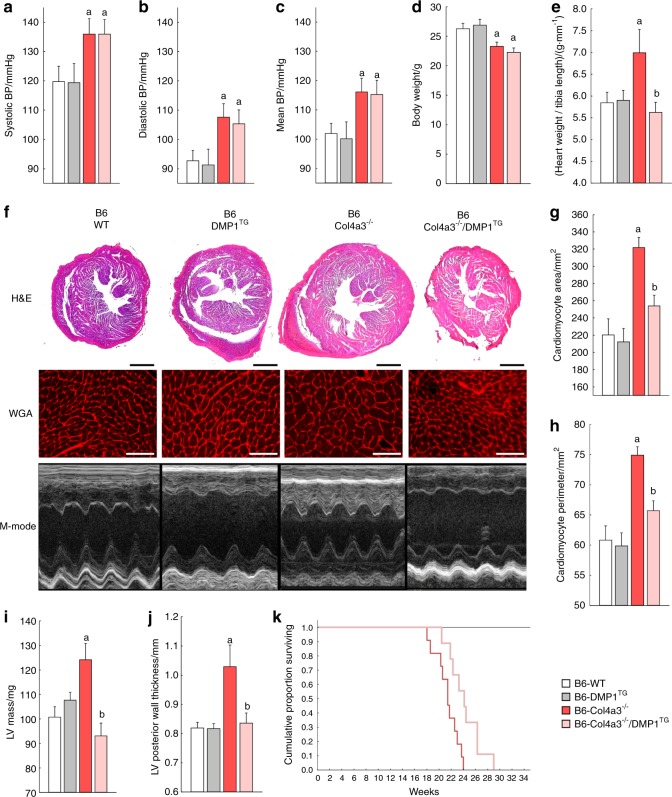
DMP1 prevents LVH and prolongs lifespan in Col4a3^−/−^ mice with advanced CKD. Blood pressure and heart morphology analyses of 20-week-old B6 WT, DMP1^TG^, Col4a3^−/−^, and Col4a3^−/−^/DMP1^TG^ mice showing: **a**–**c** Non-invasive systolic, diastolic, and mean blood pressure (BP). **d**, **e** Body weight and heart weight to tibia length ratio. **f** Bright-field microscopy of hematoxylin & eosin staining (H&E, scale bar = 1 mm) and fluorescence microscopy of wheat germ agglutinin staining (WGA, scale bar = 50 µm) of heart cross-sections, and M-mode echocardiography. **g**, **h** Cardiomyocyte cross-sectional area and perimeter calculated from WGA stained sections. **i**, **j** Left ventricular mass and left ventricular posterior wall thickness calculated from echocardiography. **k** Kaplan–Meier cumulative proportion of mice surviving. B6 WT and DMP1^TG^ mice are represented by the black line. Both B6 Col4a3^−/−^ and Col4a3^−/−^/DMP1^TG^ mice show reduced lifespan (*p* < 0.05 vs. WT), but B6 Col4a3^−/−^/DMP1^TG^ survive longer than B6 Col4a3^−/−^ mice (*P* < 0.05 vs. Col4a3^−/−^). Values are expressed as mean ± SEM; *n* ≥ 5 mice/group. *P* < 0.05 vs. ^a^ WT, ^b^ Col4a3^−/−^

Given the significant effects of DMP1 on the bone phenotype and on FGF23 production in CKD, we assessed whether correction of DMP1 depletion would have beneficial effects on the development of LVH and survival in CKD. As we previously reported,^40^ B6 Col4a3^−/−^ mice with advanced CKD are cachectic, hypertensive, develop significant LVH with preserved ejection fraction, and die prematurely (Fig. 6). Whereas ejection fraction, stroke volume and cardiac output were normal in 20-week-old B6 Col4a3^−/−^ mice compared to WT (data not shown), heart weight to tibia length ratio, cross-sectional area and perimeter of individual cardiac myocytes, LV mass and LV posterior wall thickness were all significantly increased in the B6 Col4a3^−/−^ (Fig. 6e–j). Overexpression of DMP1 in bone of B6 WT mice did not affect blood pressure, heart morphology, function or lifespan, but B6 Col4a3^−/−^/DMP1^TG^ mice showed a near complete rescue of the LVH phenotype with similar parameters of cardiac morphology to B6 WT mice despite no change in severity of hypertension (Fig. 6a–j) or kidney function, and despite worsened hyperphosphatemia (Fig. 4i, 4c). In support of the functional significance of their improved bone and cardiac phenotypes, B6 Col4a3^−/−^/DMP1^TG^ demonstrated prolonged survival compared with B6 Col4a3^−/−^ mice (24.2 ± 0.9 vs. 21.4 ± 0.6 weeks, *P* < 0.05; Fig. 6k).

## Discussion

Increased levels of circulating FGF23 is among the earliest alterations of bone and mineral metabolism that occur during progression of CKD. FGF23 is regulated by an incompletely understood interplay between systemic factors that control mineral metabolism and local bone factors that modulate turnover and mineralization, including DMP1.^18,21,23,24^ In this study, we show in the 129 Sv and B6 Col4a3^−/−^ mouse models of CKD, that DMP1 expression is reduced, and that upstream DMP1 deficiency contributes to FGF23 elevation in CKD. In prior studies using autosomal recessive hypophosphatemic rickets (ARHR) homologue DMP1^−/−^ mice, restoration of intact DMP1 or 57kDa C-terminal functional domain of DMP1 in bone successfully rescued FGF23 excess, hypophosphatemia and bone mineralization defects.^16–18^ Because it has been established that the C-terminal 57 kDa functional domain of DMP1 recapitulates the effects of intact DMP1,^17,41,42^ in this study we used 57 kDa DMP1 supplementation in Col4a3^−/−^ mice as a targeted approach to prevent FGF23 elevations and explore the downstream effects on bone, kidney and heart in a model of progressive CKD.

The Col4a3^−/−^ mice is an established model that displays many clinical features of human progressive CKD, including bone and mineral metabolism alterations, LVH when engineered on the B6 genetic background and early death.^40^ Impaired bone remodeling has been reported in previous studies in 129 Sv Col4a3^−/−^ mice.^31,37^ In this study, we further show in both genetic backgrounds, a mild bone mineralization defect, and alterations in osteocyte morphology and networks. These defects coincide with increased osteocyte apoptosis, suggesting that osteocyte apoptosis may be an underlying mechanism of osteocyte dysfunction in CKD. Similar osteocyte alterations were reported in DMP1^−/−^ mice with osteomalacia,^33^ suggesting that bone mineralization and osteocyte morphology defects observed in mice with advanced CKD may be caused, in part, by reduced DMP1 expression. The mechanisms driving DMP1 deficiency in CKD are currently unknown and will need further investigation. Regardless, using both genetic and pharmacologic approaches, DMP1 restoration corrects the bone mineralization defect, prevents alterations in osteocyte morphology and networks in mice with advanced CKD, and prevents osteocyte apoptosis in vivo and in vitro. Hyperphosphatemia, inflammation, and oxidative stress are also prominent clinical features of CKD that may contribute to osteocyte apoptosis. The anti-apoptotic role of DMP1 has been previously shown in hyperphosphatemic Klotho null mice,^20^ and in our study, cultured osteoblasts overexpressing DMP1 show lower active caspase detection at baseline and in response to the pro-inflammatory cytokine TNFα or hydrogen peroxide. This supports a direct role of DMP1 to protect osteocytes from phosphate-, inflammation-, and oxidative stress-induced apoptosis.

Consistent with the established functions of DMP1, short-term pharmacologic administration and long-term osseous overexpression of DMP1 also correct FGF23 elevations in 129 Sv and B6 Col4a3^−/−^ mice, independently of kidney disease progression and circulating levels of calcium or PTH, and despite worsening of hyperphosphatemia. This indicates that DMP1 specifically inhibits FGF23 production and that the inhibitory effects of DMP1 may supersede any direct stimulatory effects of hyperphosphatemia. Whether increased osteocyte apoptosis and impaired osteocyte morphology and connectivity contribute to increased FGF23 production remains to be determined. Nonetheless, our results reveal novel DMP1-controlled mechanisms of regulation of *Fgf23* transcription.

We have previously shown that DMP1 controls *Fgf23* transcription through local processes that involve classical paracrine FGFR1 activation, which is increased in the *Hyp* and/or *Dmp1 null* mouse models of primary FGF23 excess.^24^ More recently, NFAT signaling has emerged as an integral downstream molecular mechanism of this activation.^25^ The *Fgf23* promoter contains an NFAT response element, which controls *Fgf23* transcription in response to calcium and inflammatory stimuli.^25,38,43^ In this study, we show that *Nfat1* mRNA expression is increased in bone in CKD and that DMP1 inhibits NFAT1 signaling that is activated in CKD and prevents increases in *Fgf23* transcription. Therefore, NFAT signaling represents the first direct link between DMP1 and *Fgf23* transcription in bone. However, DMP1 rescues *Fgf23* transcription only in mice with early CKD resulting in complete correction of early circulating FGF23 elevations. In contrast, DMP1 does not rescue *Fgf23* transcription in mice with advanced CKD, suggesting that other stimuli, such as elevated PTH and chronic inflammation eventually override these effects. In DMP1-treated mice with advanced CKD, the dramatic elevations of circulating FGF23 levels are partially corrected despite increased and unchanged *Fgf23* transcription, which suggests that DMP1 may also regulate FGF23 post-translational processing in CKD.

Elevations of circulating FGF23 levels during CKD progression are independently associated with cardiovascular mortality, possibly via direct and potentially reversible effects of FGF23 on cardiac myocytes that culminates in LVH.^11–15^ Indeed, mortality due to cardiovascular disease is extremely high among patients with CKD.^7,8,10^ However, the direct role of FGF23 in CKD-associated cardiac hypertrophy is currently under debate. While studies using conditional FGF23 deletion or specific FGF23 blocking antibodies would theoretically be ideal for establishing or refuting a direct role of FGF23 in LVH, these studies are complicated by the profound alterations in mineral metabolism that occur when FGF23 effects are fully eliminated or neutralized.^44^ We previously showed that B6 Col4a3^−/−^ mice with slow CKD progression display LVH at 20 weeks of age, and die a few weeks later.^29^ By overexpressing DMP1 in the bones of B6 Col4a3^−/−^ mice, we now present a model of CKD in which FGF23 levels are partially lowered, while all other features of advanced CKD, including impaired kidney function, altered mineral metabolism, and hypertension, are worsened or conserved, yet LVH improves markedly. Therefore, the present study is the first to show that lowering FGF23 levels in a CKD model can attenuate development of LVH.

Importantly, despite elevated blood pressure, cardiac function remains normal in mice with advanced CKD and significant LVH, and the correction of LVH with DMP1 does not affect cardiac function or blood pressure. This suggests that LVH may not be an adaptive response of cardiac remodeling to preserve ejection fraction and prevent heart failure in CKD. In contrast, correction of FGF23 and LVH by DMP1 in CKD is associated with prolonged survival, supporting the possible important contribution of FGF23 and LVH to increased mortality in CKD. While our results may be specific to the Alport’s model of CKD, they suggest the potential benefit of partially lowering FGF23 in CKD, for example with low or intermediate doses of anti-FGF23 antibodies, even at the cost of a modest increase in serum phosphate, which could be mitigated with concomitant therapies and tested in innovative randomized clinical trials. Additional studies testing anti-FGF23 antibodies and DMP1 in additional models of CKD will be needed to fully establish the beneficial effects of preventing FGF23 elevations in CKD.

**Fig. 7:**
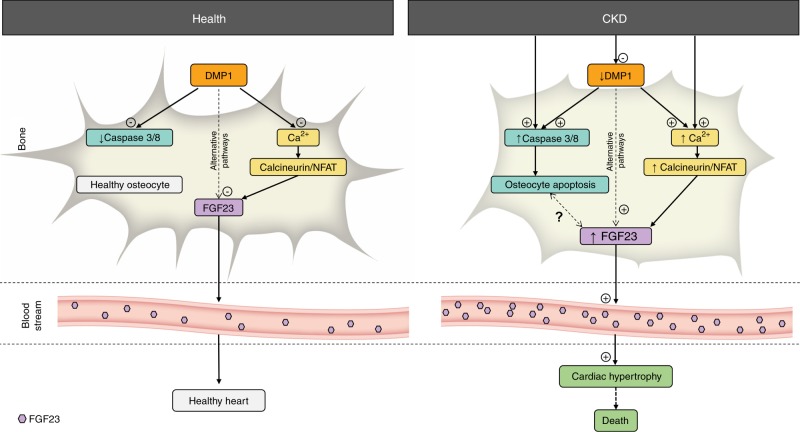
Protective role of DMP1 through regulation of apoptosis and calcineurin/NFAT signaling in osteocytes. DMP1 and FGF23 are both mainly produced and secreted by osteocytes. In health, DMP1 maintains osteocyte networks integrity by maintaining adequate bone mineralization and preventing osteocyte apoptosis. DMP1 also inhibits FGF23 transcription in osteocytes which contributes to low baseline levels of circulating FGF23. Mice with advanced CKD show reduced DMP1 expression, which contributes to increased caspase 3/8 and calcineurin/NFAT signaling, resulting in increased osteocyte apoptosis, altered osteocyte morphology and networks, and increased production of FGF23. Elevated levels of circulating FGF23 promote left ventricular hypertrophy and premature death. Consequently, DMP1 supplementation in mice with CKD prevents osteocyte apoptosis, improves osteocyte morphology and connectivity, prevents FGF23 elevation, protects against the development of cardiovascular disease and improves survival

To conclude, our data show that restoring DMP1 in CKD improves bone mineralization, protects osteocytes from apoptosis, and preserves the integrity of osteocyte networks. These effects, combined with DMP1 inhibitory effects on FGF23 transcription, lead to less severe elevations of circulating FGF23 levels and positive effects on cardiac health and survival (Fig. 7), despite persistent kidney disease, hypertension, hyperphosphatemia, and hyperparathyroidism. Thus, our data support a potential therapeutic role for DMP1 in CKD to reduce FGF23 and potentially attenuate bone and heart disease.

## Materials and methods

### In vivo studies

#### Study approval

All animal studies were conducted in accordance with the Northwestern University Institutional Animal Care and Use Committee.

#### Genetic overexpression of DMP1

C57Bl6/J mice expressing a transgene containing a truncated DMP1 sequence downstream of a Collagen Type I 3.6Kb promoter producing the C-terminal 57 kDa functional domain of DMP1 (DMP1^TG^) were generously provided by Dr. Feng (TXA&M University, Dallas, TX).^42^ We purchased 129X1/SvJ (129 Sv) Col4a3^−/−^ mice from The Jackson Laboratories (Bar Harbor, ME, USA) and outcrossed 129 Sv Col4a3 heterozygous with C57Bl6/J wild-type (WT) mice for three generations (N3). We crossed C57Bl6/J(N3)-Col4a3 heterozygotes with DMP1^TG^ mice and further crossed the F1 transgenic heterozygotes to generate C57Bl6/J(N4) WT, DMP1^TG^, Col4a3^−/−^, and Col4a3^−/−^/DMP1^TG^ mice that contained 94% C57Bl6/J genome. We maintained this newly created strain separately for more than five generations.^40^ We harvested samples on a set of 20–23-week-old male littermates. We recorded body weight at sacrifice. In a separate set of animals, we recorded the age of death on ten Col4a3^−/−^ and Col4a3^−/−^/DMP1^TG^ male littermates to assess effects on lifespan.

#### IP injections

We purchased pure 129 Sv Col4a3^−/−^ mice from The Jackson Laboratories (Bar Harbor, ME, USA) and maintained them on the 129 Sv genetic background for over ten generations. For pharmacologic administration of DMP1, we injected 10 ng·g^–1^ per day of mouse recombinant His-tagged C-terminal DMP1 (R&D Systems, Minneapolis, MN) or normal saline control to 5-week-old (early CKD) and 8-week-old (advanced CKD) pure 129 Sv Col4a3^−/−^ mice and WT mice once a day for seven days. We harvested samples on 6- and 9-week-old male littermates. For calcium administration, we performed a single 10 μL·g^–1^ injection of 3% calcium chloride solution or normal saline control^22,37^ to 12-week-old WT and DMP1^TG^ littermate mice. We harvested samples 6 h post-injection. For NFAT inhibitor administration, we injected animals twice, 18 h and 6 h prior to sacrifice with a solution of 10 mg·kg^–1^ of 11R-VIVIT cell permeable NFAT inhibitor (Cat# 480401, Millipore Sigma, Burlington, MA, USA). Mice were co-injected with saline (Ctr) or 10 μL·g^–1^ injection of 3% calcium chloride solution.

#### Blood pressure

We recorded blood pressure in sentient mice using a computerized mouse tail-cuff system (CODA, Kent Scientific, Torrington, CT). We acquired readings for 20 cycles, once a day during three consecutive days to acclimate each mouse to the system and reduce environmental stress. We analyzed the third-day data from habituated mice.

#### Echocardiography

We performed echocardiography under inhalant isoflurane anesthesia 1 week prior to sacrifice using a Vevo 770 High-Resolution In vivo Micro-Imaging System (VisualSonics, Toronto, Canada). We used the parasternal short- and long-axis views to obtain 2-dimensional and M-mode images. We acquired at least 10 independent cardiac cycles for each experiment.

### Ex vivo imaging

#### 3D microtomography

We scanned whole femurs with µCT40 (Scanco Medical, Brüttisellen, Switzerland) at 10μm isotropic voxel size, energy level of 55 keV, and intensity of 145 μA.^37^ The trabecular bone structure was analyzed within 1 mm of the secondary spongiosa of the distal femur underneath the growth plate. The cortical bone structure was analyzed within 1 mm at the midshaft of each femur. All gray-scale images were segmented using a fixed Gaussian filter and threshold for all data.

#### Histomorphometry and histology

We injected mice with Alizarin Red S at 7 and 2 days prior to harvest for dynamic histomorphometry measurements. We measured femurs, tibiae and hearts using a slide caliper and weighted prior to fixation. We normalized whole heart weight to tibia length to account for growth variability. We fixed and dehydrated femurs, tibiae and hearts in ethanol, we embedded femurs in methylmetacrylate (MMA), tibiae and hearts in paraffin and we cut non-serial 5-μm MMA and paraffin slices (Leica Microsystems Inc., Buffalo Grove, IL) for downstream histological and immunohistochemistry analyzes. We captured bright-field and fluorescence microscopy images (Leica Microsystems, Buffalo Grove, IL, USA). For bone histology, we used unstained longitudinal femoral sections, modified trichrome Goldner stained sections and tartrate-resistant acidic phosphatase (TRAcP) activity stained sections according to previously described methods.^45^ For analysis of the cardiac phenotype we used cross sections from the mid-chamber of the heart. We stained the sections with hematoxylin and eosin (H&E) to determine cardiac morphology and with Alexa Fluor 594 wheat germ agglutinin (WGA) conjugate to determine cardiomyocyte cross-sectional area. We calculated the cardiomyocyte surface area and perimeter using Image J software (National Institutes of Health, Bethesda, MD) on five fields from four randomly selected heart sections (×20 magnification).

#### TUNEL and Immunohistochemistry

We used longitudinal tibia sections for TUNEL and immunostaining. We deparaffinized, rehydrated and incubated the sections in citric acid buffer (10 mmol·L^–1^, pH 3) for 60 min at 37 °C (Vector Labs, Burlingame, CA) for antigen retrieval, and 20 min in 1X animal-free blocker (Vector Labs, Burlingame, CA) prior to specific stainings. For detection of endogenous DMP1 in cortical bone, we incubated the sections with anti-DMP1 primary antibody (#ab103203, C-terminal region, Abcam, Cambridge, MA, USA) for 1 h. For detection of injected His-tagged recombinant DMP1 in cortical bone, we incubated sections with anti-His tag primary antibody (Abcam, Cambridge, MA) for 1 h. We then used the immunohistological Vectastain ABC kit (Vector Labs, Burlingame, CA) and performed detection by bright-field microscopy (Leica Microsystems, Buffalo Grove, IL, USA). We performed TUNEL staining using ApopTag Peroxidase In Situ Apoptosis Detection Kit according to manufacturer’s protocol (Millipore Corporation, Temecula, CA) and quantified the ratio of TUNEL-positive osteocytes to total osteocytes on three separate sections per animal.

#### Acid-etched scanning electron microscopy (SEM)

We embedded tibia samples in MMA, and acid-etched the polished surface with 37% phosphoric acid for 2–10 s, washed twice with water, followed by 5% sodium hypochlorite for 5 min, and washed again in water. We coated the air-dried samples with gold and palladium, and examined by FEI/Philips XL30 Field emission environmental SEM according to previously described protocol.^21^

#### FITC-Imaris

We rinsed mouse tibia in PBS, fixed in 70% ethanol for 2 days at room temperature, slow dehydrated in 95% ethanol for 1 day and in 100% ethanol for 1 more day. We stained the samples with 1% FITC (Sigma, St. Louis, MO, cat. no. F7250) in 100% ethanol overnight, followed by continuous dehydration with 100% ethanol for 1 more day, and acetone for 2 days. We then embedded the samples in MMA. We kept the samples away from light during the entire procedure. We cut the specimen to cubic size by diamond saw for further dehydration. We sectioned the embedded plastic blocks into ~1–2-mm-thick slices using a water-cooled diamond-impregnated circular saw (Isomet, Buehler, Germany). We further sanded these slices down to 5 100 mm thickness using 6 grades (80, 200, 400, 600, 800, and 1 200 grit) of sanding papers, and polished on a soft cloth rotating wheel with 1-mm alumina alpha micropolish II solutions (Buehler, no. 406323016). After polishing, we immersed the slides in a water-soluble mounting medium for confocal imaging and then covered with a plastic cover. We performed all sample preparation, staining, imaging, and Autoquant and Imaris analyses according to previously described protocol.^33^

### Quantitative RT-PCR

We isolated total RNA from heart, kidney and tibia samples harvested at sacrifice and from primary osteoblasts cultures using TRI reagent and synthetized first-strand cDNA (iScript cDNA Synthesis Kit, Bio-Rad Laboratories, Hercules, CA). We used the iCycler iQ real-time PCR detection system, iQ SYBR Green supermix (Bio-Rad Laboratories, Hercules, CA) and adequate primer pairs (Table S1) for real-time quantitative PCR analysis. The threshold of detection of each gene expression was set at optimal reaction efficiency. The expression was plotted against a standard dilution curve of relative concentration, normalized to glyceraldehyde-3-phosphate dehydrogenase (GAPDH) expression in the same sample and expressed as fold change versus wild-type.

### In vitro studies

#### Cell cultures

We cultured MC3T3-E1 osteoblastic cell lines (ATCC) according to American Type Culture Collection guidelines. We prepared bone marrow stromal cells (BMSCs) from 8-week-old WT and DMP1^TG^ mice according to a previously described protocol.^46^ We maintained MC3T3-E1 and BMSCs in α-MEM containing 10% FBS, 10 U·mL^–1^ penicillin, and 100 μg·mL^–1^ streptomycin. For all experimental conditions, we plated MC3T3-E1 at 3 × 10^4^ cells per well and BMSCs at 10 × 10^4^ cells per well and cultured for 3 weeks in osteoblast-differentiating medium (α-minimal essential medium, 10% fetal bovine serum, 10 U·mL^–1^ penicillin, 100 μg·mL^–1^ streptomycin, 10 mmol·L^–1^ β-glycerophosphate, and 50 μg·mL^–1^ ascorbic acid; Sigma–Aldrich, St Louis, MO) prior to treatment and collection. To assess *Fgf23* promoter activity, MC3T3-E1 cells were stably transfected with the pLuc-*Fgf23* promoter or a mutated NFAT response element pLuc-Fgf23 plasmids carrying a secreted luciferase expression cassette under the control of the proximal *Fgf23* promoter, a secreted alkaline phosphatase (SEALP) under the control of the CMV promoter, and a puromycin resistance cassette (Genecopoeia, Rockville, MD).

#### Cell treatments and assays

To measure *Fgf23* promoter activity, we placed the cells in calcium-free Optimem medium containing 1% FBS 10 U·mL^–1^ penicillin and 100 μg·mL^–1^ streptomycin, supplemented with 0, 6, or 12 mmol·L^–1^ calcium for the last 12 h of culture. We co-treated the cells with 0, 1, and 10 μg·mL^–1^ of NFAT inhibitor (Cat# 480401, Millipore Sigma, Burlington, MA, USA) or with 0, 10, 50, and 100 ng·mL^–1^ of C-terminal DMP1 protein, synthetized and purified by Northwestern Protein Core. For promoter activity experiments, we collected cell culture media after 6 and 12 h during the last day of culture. We performed luciferase activity assays in duplicate according to the manufacturer's instructions (Genecopoeia, Rockville, MD). Promoter activity is represented by relative luciferase unit normalized to pSEALP-CMV control. We conducted all experiments in triplicate.

### Serum and urine biochemistry

We collected overnight urine samples from fasted animals housed in metabolic cages and serum samples by intracardiac exsanguination. We measured intact FGF23 levels using a murine iFGF23 ELISA that measures the intact active protein exclusively, and total FGF23 using the cFGF23 ELISA that recognizes the full-length protein and its C-terminal cleavage fragments (both from Immutopics, Carlsbad, CA). We measured serum PTH using a mouse intact ELISA (Immutopics, Carlsbad, CA), serum 1,25(OH)_2_D by immunoassay (Immunodiagnostic Systems, Gaithersburg, MD), and serum and urine calcium, phosphate, blood urea nitrogen, and creatinine using colorimetric assays (Pointe Scientific, Canton, MI).

### Statistics

Data are presented as mean ± SEM. We used one-way ANOVA followed by *post hoc* *t*-tests to test statistical differences (Statistica software, Statsoft, Tulsa, OK). Differences were considered statistically significant at *P* values <0.05.

## Supplementary information

Supplemental Material clean version

Figure S1

Figure S2

Figure S3

Figure S4

Figure S5

## References

[1] Wolf M (2010). Forging forward with 10 burning questions on FGF23 in kidney disease. J. Am. Soc. Nephrol..

[2] Malluche HH, Mawad H, Monier-Faugere MC (2004). The importance of bone health in end-stage renal disease: out of the frying pan, into the fire?. Nephrol. Dial. Transplant..

[3] Shimada T (2004). Targeted ablation of Fgf23 demonstrates an essential physiological role of FGF23 in phosphate and vitamin D metabolism. J. Clin. Invest..

[4] Shimada T (2004). FGF-23 is a potent regulator of vitamin D metabolism and phosphate homeostasis. J. Bone Miner. Res..

[5] Isakova T (2011). Fibroblast growth factor 23 is elevated before parathyroid hormone and phosphate in chronic kidney disease. Kidney Int..

[6] Wolf M (2012). Update on fibroblast growth factor 23 in chronic kidney disease. Kidney Int..

[7] Gutierrez OM (2008). Fibroblast growth factor 23 and mortality among patients undergoing hemodialysis. N. Engl. J. Med..

[8] Isakova T (2018). Longitudinal FGF23 trajectories and mortality in patients with CKD. J. Am. Soc. Nephrol..

[9] Scialla JJ (2014). Fibroblast growth factor-23 and cardiovascular events in CKD. J. Am. Soc. Nephrol..

[10] Isakova T (2011). Fibroblast growth factor 23 and risks of mortality and end-stage renal disease in patients with chronic kidney disease. JAMA.

[11] Gutierrez OM (2009). Fibroblast growth factor 23 and left ventricular hypertrophy in chronic kidney disease. Circulation.

[12] Faul C (2011). FGF23 induces left ventricular hypertrophy. J. Clin. Invest..

[13] Grabner A (2015). Activation of cardiac fibroblast growth factor receptor 4 causes left ventricular hypertrophy. Cell Metab..

[14] Leifheit-Nestler M (2016). Induction of cardiac FGF23/FGFR4 expression is associated with left ventricular hypertrophy in patients with chronic kidney disease. Nephrol. Dial. Transplant..

[15] Mitsnefes MM (2018). FGF23 and Left Ventricular Hypertrophy in Children with CKD. Clin. J. Am. Soc. Nephrol..

[16] Sun Y (2010). Failure to process dentin matrix protein 1 (DMP1) into fragments leads to its loss of function in osteogenesis. J. Biol. Chem..

[17] Lu Y (2011). The biological function of DMP-1 in osteocyte maturation is mediated by its 57-kDa C-terminal fragment. J. Bone Miner. Res..

[18] Martin A (2012). Overexpression of the DMP1 C-terminal fragment stimulates FGF23 and exacerbates the hypophosphatemic rickets phenotype in Hyp mice. Mol. Endocrinol..

[19] He G, Dahl T, Veis A, George A (2003). Nucleation of apatite crystals in vitro by self-assembled dentin matrix protein 1. Nat. Mater..

[20] Rangiani A (2012). Protective roles of DMP1 in high phosphate homeostasis. PLoS ONE..

[21] Feng JQ (2006). Loss of DMP1 causes rickets and osteomalacia and identifies a role for osteocytes in mineral metabolism. Nat. Genet..

[22] Liu S (2008). Pathogenic role of Fgf23 in Dmp1-null mice. Am. J. Physiol. Endocrinol. Metab..

[23] Lorenz-Depiereux B (2006). DMP1 mutations in autosomal recessive hypophosphatemia implicate a bone matrix protein in the regulation of phosphate homeostasis. Nat. Genet..

[24] Martin A (2011). Bone proteins PHEX and DMP1 regulate fibroblastic growth factor Fgf23 expression in osteocytes through a common pathway involving FGF receptor (FGFR) signaling. FASEB J..

[25] Han X, Xiao Z, Quarles LD (2015). Membrane and integrative nuclear fibroblastic growth factor receptor (FGFR) regulation of FGF-23. J. Biol. Chem..

[26] Pereira RC (2009). Patterns of FGF-23, DMP1, and MEPE expression in patients with chronic kidney disease. Bone.

[27] Santos MFP (2018). Comparison of clinical, biochemical and histomorphometric analysis of bone biopsies in dialysis patients with and without fractures. J. Bone Miner. Metab..

[28] Yoon CY (2016). Low dentin matrix protein 1 is associated with incident cardiovascular events in peritoneal dialysis patients. J. Bone Miner. Res..

[29] Neuburg S (2017). Genetic background influences cardiac phenotype in murine chronic kidney disease. Nephrol. Dial. Transplant..

[30] Cosgrove D (1996). Collagen COL4A3 knockout: a mouse model for autosomal Alport syndrome. Genes & development.

[31] Stubbs JR (2012). Longitudinal evaluation of FGF23 changes and mineral metabolism abnormalities in a mouse model of chronic kidney disease. J. Bone Miner. Res..

[32] Cosgrove D, Kalluri R, Miner JH, Segal Y, Borza DB (2007). Choosing a mouse model to study the molecular pathobiology of Alport glomerulonephritis. Kidney Int..

[33] Ren Y, Lin S, Jing Y, Dechow PC, Feng JQ (2014). A novel way to statistically analyze morphologic changes in Dmp1-null osteocytes. Connect. Tissue Res..

[34] Tan SD (2006). Fluid shear stress inhibits TNFalpha-induced osteocyte apoptosis. J. Dent. Res..

[35] Kikuyama A (2002). Hydrogen peroxide induces apoptosis of osteocytes: involvement of calcium ion and caspase activity. Calcif. Tissue Int..

[36] Dai B (2012). A comparative transcriptome analysis identifying FGF23 regulated genes in the kidney of a mouse CKD model. PLoS ONE..

[37] David V (2013). Calcium regulates FGF-23 expression in bone. Endocrinology.

[38] Liu S (2006). Fibroblast growth factor 23 is a counter-regulatory phosphaturic hormone for vitamin D. J. Am. Soc. Nephrol..

[39] David V (2016). Inflammation and functional iron deficiency regulate fibroblast growth factor 23 production. Kidney Int..

[40] Neuburg S (2018). Genetic background influences cardiac phenotype in murine chronic kidney disease. Nephrol. Dial. Transplant..

[41] Lu Y, Qin C, Xie Y, Bonewald LF, Feng JQ (2009). Studies of the DMP1 57-kDa Functional Domain both in vivo and in vitro. Cells Tissues Organs.

[42] Lu Y (2007). Rescue of odontogenesis in Dmp1-deficient mice by targeted re-expression of DMP1 reveals roles for DMP1 in early odontogenesis and dentin apposition in vivo. Dev. Biol..

[43] David V, Francis C, Babitt JL (2017). Ironing out the cross talk between FGF23 and inflammation. Am. J. Physiol. Renal. Physiol..

[44] Shalhoub V (2012). FGF23 neutralization improves chronic kidney disease-associated hyperparathyroidism yet increases mortality. J. Clin. Invest..

[45] Martin A (2005). Leptin modulates both resorption and formation while preventing disuse-induced bone loss in tail-suspended female rats. Endocrinology.

[46] Martin A (2008). Degradation of MEPE, DMP1, and release of SIBLING ASARM-peptides (minhibins): ASARM-peptide(s) are directly responsible for defective mineralization in HYP. Endocrinology.

