# Supplementary material for: DMP1 prevents osteocyte alterations, FGF23 elevation and left ventricular hypertrophy in mice with chronic kidney disease
Source: Bone Res. 2019 Apr 25;7:12. doi: 10.1038/s41413-019-0051-1 (PMC6483996; doi:10.1038/s41413-019-0051-1)
Supplement: Supplementary file 1 — Supplemental Material clean version [file 41413_2019_51_MOESM1_ESM.docx]

# SUPPLEMENTAL METHODS

## Mineralization

We prepared bone marrow stromal cells (BMSCs) from 18 week-old B6 WT, DMP1^TG^, Col4a3^-/-^ and Col4a3^-/-^/DMP1^TG^ mice, stained with Alizarin Red S and quantified staining following a previously described protocol ^1^.

## Apoptosis

To assess DMP1 impact on cell apoptosis, MC3T3-E1 cells were stably transfected with either the Dmp1 ORF (NM_016779.2) under the control of the CMV promoter (p[CMV/DMP1]) and a puromycin resistance cassette (Genecopoeia, Rockville, MD) or an empty vector (Ctr).

We induced apoptosis in Ctr and DMP1-transfected MC3T3-E1 osteoblasts by treatment with 0, 5, 10 and 50 ng/mL TNFα (Sigma-Aldrich, Saint Louis, MO) for the last 18 hours of culture or with 0, 0.3, 0.6 and 1.2mM of hydrogen peroxide for the last hour of culture. After treatment, we washed the cells with fresh differentiation medium and stained using the Image-iT ™ LIVE Red Caspase-3 and -7 Detection Kit (Invitrogen) following manufacturer’s recommended protocol. We imaged the cells using fluorescent microscopy (Leica Microsystems Inc., Buffalo Grove, IL) and calculated the ratio of caspase staining to total cell count on 5 randomly selected fields per well and 6 wells per condition using ImageJ software (NIH, Bethesda, MD).

## Kidney histology

Left kidneys were harvested at sacrifice and fixed in 100% ethanol. Kidneys were embedded in paraffin and were cut into 5-µm-thick longitudinal kidney sections using a rotary microtome (Leica Microsystems Inc., Buffalo Grove, IL). Kidney sections were stained with H&E to determine renal glomerular and tubular morphology and with picrosirius red (PSR) to determine renal fibrosis.

## DMP1 overexpression in 129Sv mice

We crossed pure 129Sv Col4a3 heterozygotes with pure C57Bl6/J DMP1^TG^ mice and further crossed the F1 transgenic heterozygotes to pure 129Sv Col4a3 heterozygotes to generate WT, DMP1^TG^, Col4a3^-/-^ and Col4a3^-/-^/DMP1^TG^ mice containing 75% 129Sv and 25% B6 genomes. We maintained this newly created strain separately for more than five generations as previously described ^2^. We harvested samples on a set of 10 week-old male littermates.

# SUPPLEMENTAL FIGURES AND FIGURE LEGENDS


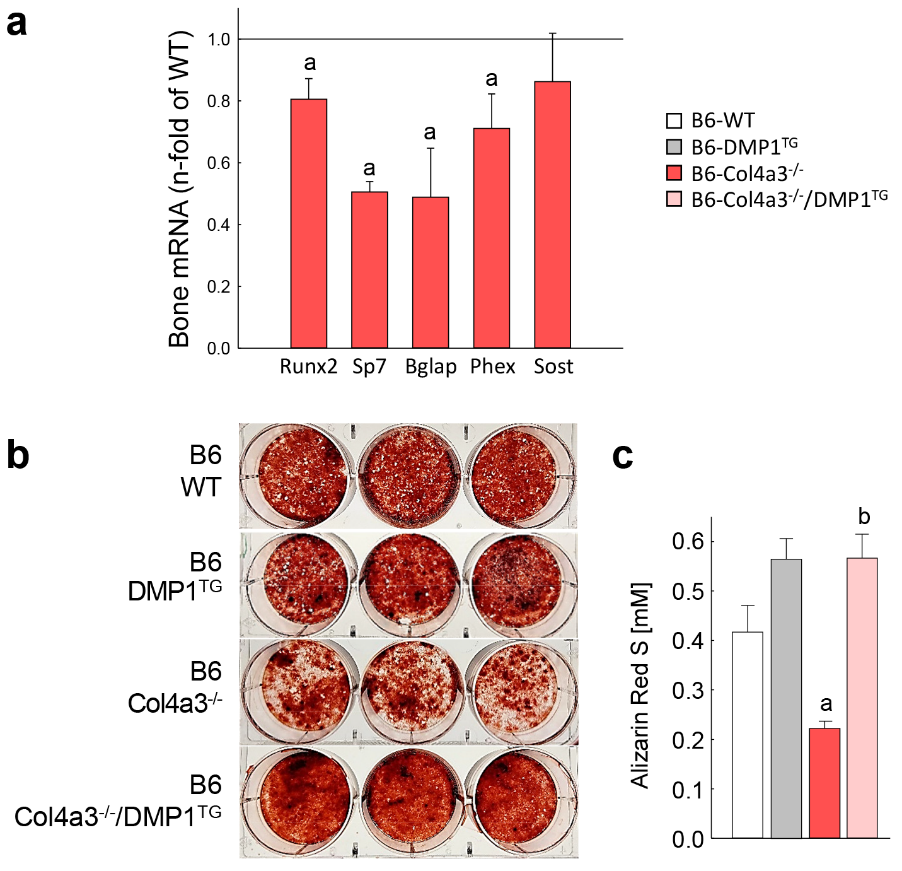


## **Figure S1: Altered osteoblast differentiation and mineralization in CKD**

(a) Reduced mRNA expression of osteoblast and osteocyte markers in whole bone of B6 Col4a3^KO^ mice. (b) Alizarin Red S staining of primary osteoblasts isolated from bones of B6 WT, DMP1^TG^, Col4a3^KO^ and Col4a3^KO^/DMP1^TG^ mice and (c) quantification showing matrix mineralization is reduced in B6 Col4a3^KO^ and increased in Col4a3^KO^/DMP1^TG^.

*Values are expressed as mean ± SEM; n = 3-12 / group. p< 0. 05 vs. ^a^ WT, ^b^ Col4a3^KO^.*


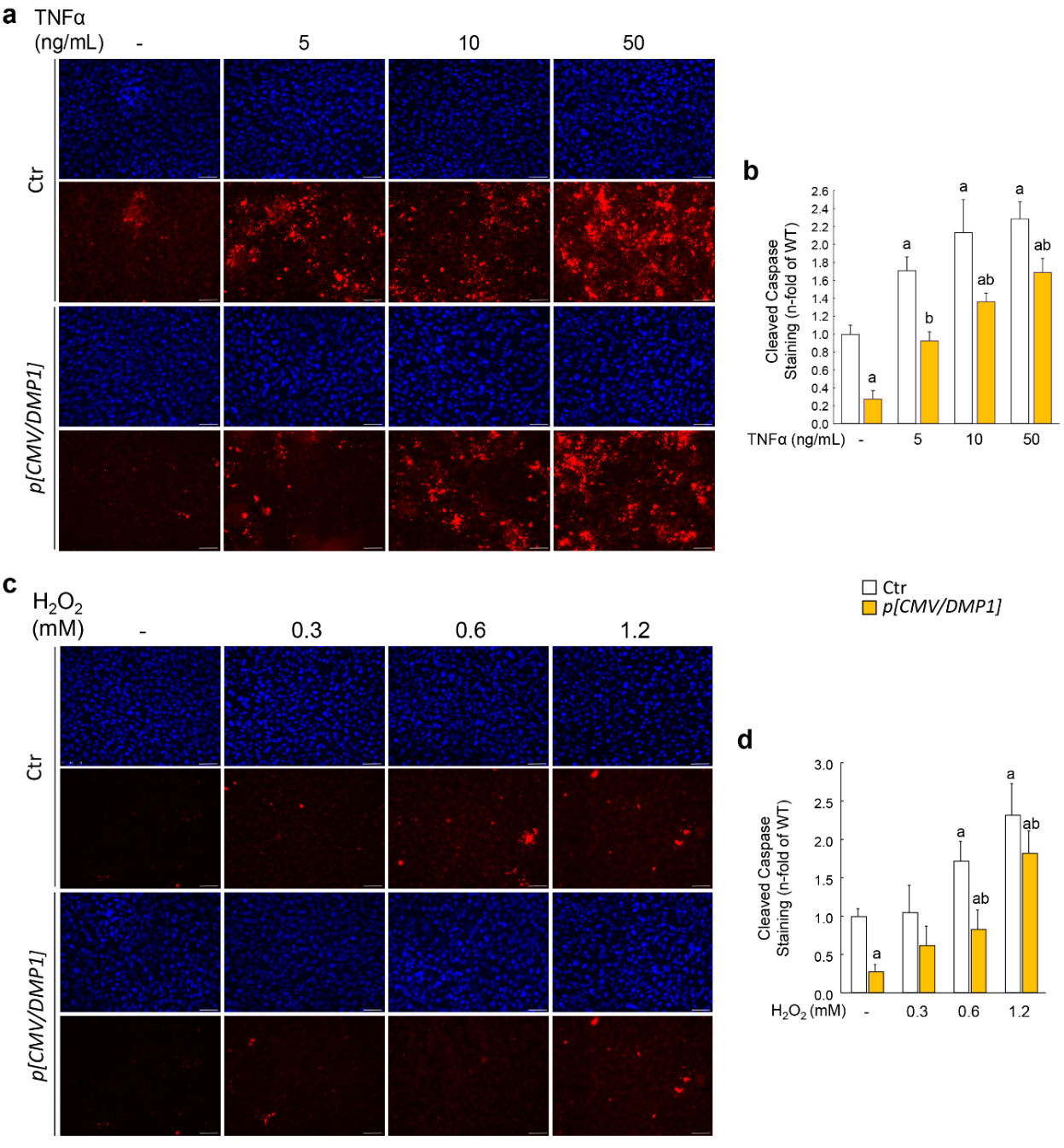


## **Figure S2: DMP1 prevents osteocyte apoptosis**

Fluorescent microscopy of Image-iT ™ LIVE Red Caspase and DAPI staining of non-transfected (Ctr) and DMP1-transfected (p[CMV/DMP1]) MC3T3-E1 osteoblasts treated with increasing doses of TNFα (**a**) or hydrogen peroxide (**c**) and respective quantifications of active caspase staining positive area to total cell count (**b, d**).

*Values are expressed as mean ± SEM; n > 3 / group. p< 0. 05 vs. ^a^ WT.*


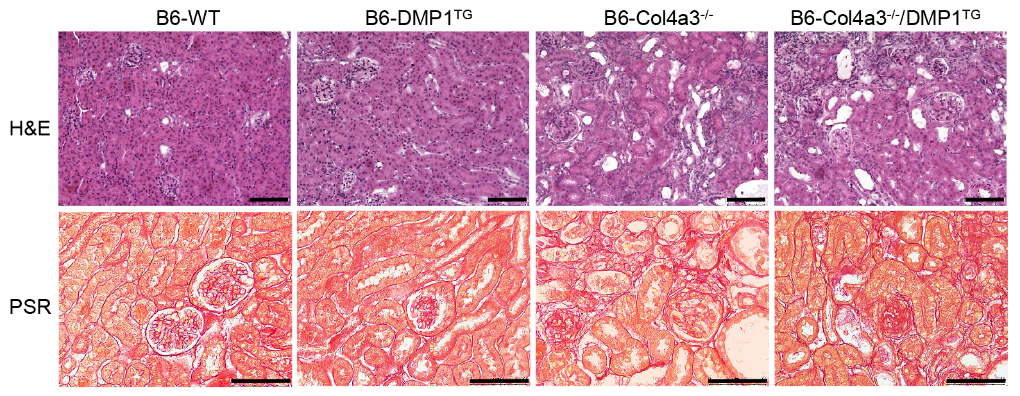


## Figure S3: Altered kidney morphology in mice with advanced CKD

Bright field microscopy of hematoxylin & eosin staining (H&E, scale bar = 100µm) and picrosirius red staining (PSR, scale bar = 75µm) of kidneys from 20 week-old B6 WT, DMP1^TG^, Col4a3^KO^ and Col4a3^KO^/DMP1^TG^ mice showing tubular atrophy, glomerulosclerosis and interstitial fibrosis in both B6 Col4a3^KO^ and Col4a3^KO^/DMP1^TG^ groups.

**
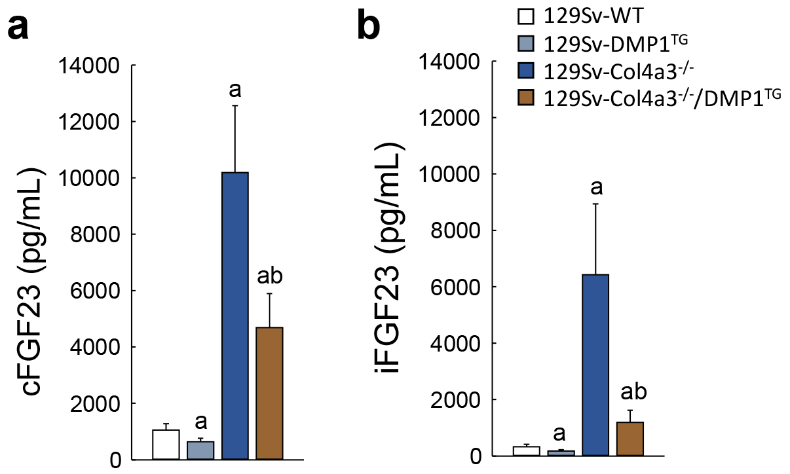
**

## Figure S4: DMP1 reduces serum FGF23 levels in CKD

Serum levels of (**a**) total FGF23 (cFGF23) and (**b**) intact FGF23 (iFGF23) in 129Sv WT, DMP1^TG^, Col4a3^KO^ and Col4a3^KO^/DMP1^TG^ mice with advanced CKD.

*Values are expressed as mean ± SEM; n ≥ 7 / group. p< 0.05 vs. ^a^ WT, ^b^ Col4a3^KO^.*

*
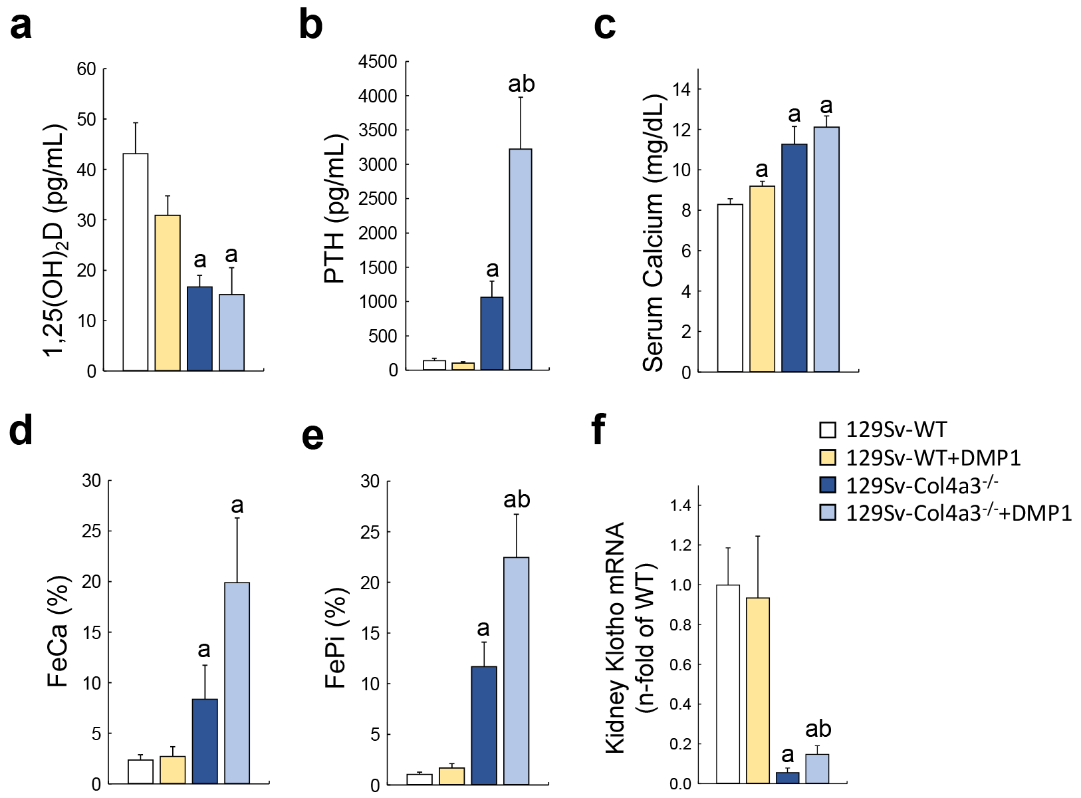
*

## Figure S5: Altered mineral metabolism in mice with advanced CKD

Serum and urine biochemistry analysis of 9 week-old 129Sv WT and Col4a3^KO^ treated with mouse recombinant DMP1 or saline control for one week.

(**a-b**) Serum levels of parathyroid hormone (PTH) and 1,25(OH)_2_D.

(**c**) Serum calcium levels.

(**d-e**) Fractional excretions of calcium (FeCa) and phosphate (FePi).

(**f**) Klotho mRNA expression in the kidney.

*Values are expressed as mean ± SEM; n ≥ 7 / group. p< 0.05 vs. ^a^ WT, ^b^ Col4a3^KO^.*

## Table S1: qPCR primers

|  | Forward primer | Reverse primer |
| --- | --- | --- |
| GAPDH | AATGGGGTGAGGCCGGTGCT | GCAGTGATGGCATGGACTGTGGT |
| Runx2 | CGGACGAGGCAAGAGTTTCA | GGATGAGGAATGCGCCCTAA |
| Sp7 | TCTCAAGCACCAATGGACTCC | CCAGGAAATGAGTGAGGGAAGG |
| Bglap | CCGCCTACAAACGCATCTATG | GCTGCTGTGACATCCATACTTG |
| Phex | GTGGTGGTCTGTGGAATCAG | AGCCGGCTTTCTTCCAATA |
| Sost | GTGCCTCATCTGCCTACTTGT | CGGACACATCTTTGGCGTCAT |
| Dmp1 | AGTGAGGAGGACAGCCTGAA | GAGGCTCTCGTTGGACTCAC |
| αKlotho | CATAGGGGCTACAGCATCCG | TTCTTGGCTACAACCCCGTC |
| FGF23 | CACTGCTAGAGCCTATCC | CACTGTAGATGGTCTGATGG |
| NFATc1 | CCACGTCTGGGAGATGGAAG | GGGGGCTGCTCAGTAAAAAC |

*GAPDH: glyceraldehyde 3-phosphate dehydrogenase; Runx2: Runt related transcription factor 2; Sp7: Osterix; Bglap: Bone gamma-carboxyglutamic acid-containing protein (osteocalcin); Phex: Phosphate regulating gene with homologies to endopeptidases on the X chromosome; NFATc1: Nuclear factor of activated T-cells cytoplasmic 1.*

# REFERENCES

1. Martin A, David V, Laurence JS, Schwarz PM, Lafer EM, Hedge AM, Rowe PS. Degradation of mepe, dmp1, and release of sibling asarm-peptides (minhibins): Asarm-peptide(s) are directly responsible for defective mineralization in hyp. *Endocrinology*. 2008;149:1757-1772

2. Neuburg S, Dussold C, Gerber C, Wang X, Francis C, Qi L, David V, Wolf M, Martin A. Genetic background influences cardiac phenotype in murine chronic kidney disease. *Nephrol Dial Transplant*. 2018;33:1129-1137
