# Supplementary figures and images for: DMP1 prevents osteocyte alterations, FGF23 elevation and left ventricular hypertrophy in mice with chronic kidney disease
Source: Bone Res. 2019 Apr 25;7:12. doi: 10.1038/s41413-019-0051-1 (PMC6483996; doi:10.1038/s41413-019-0051-1)

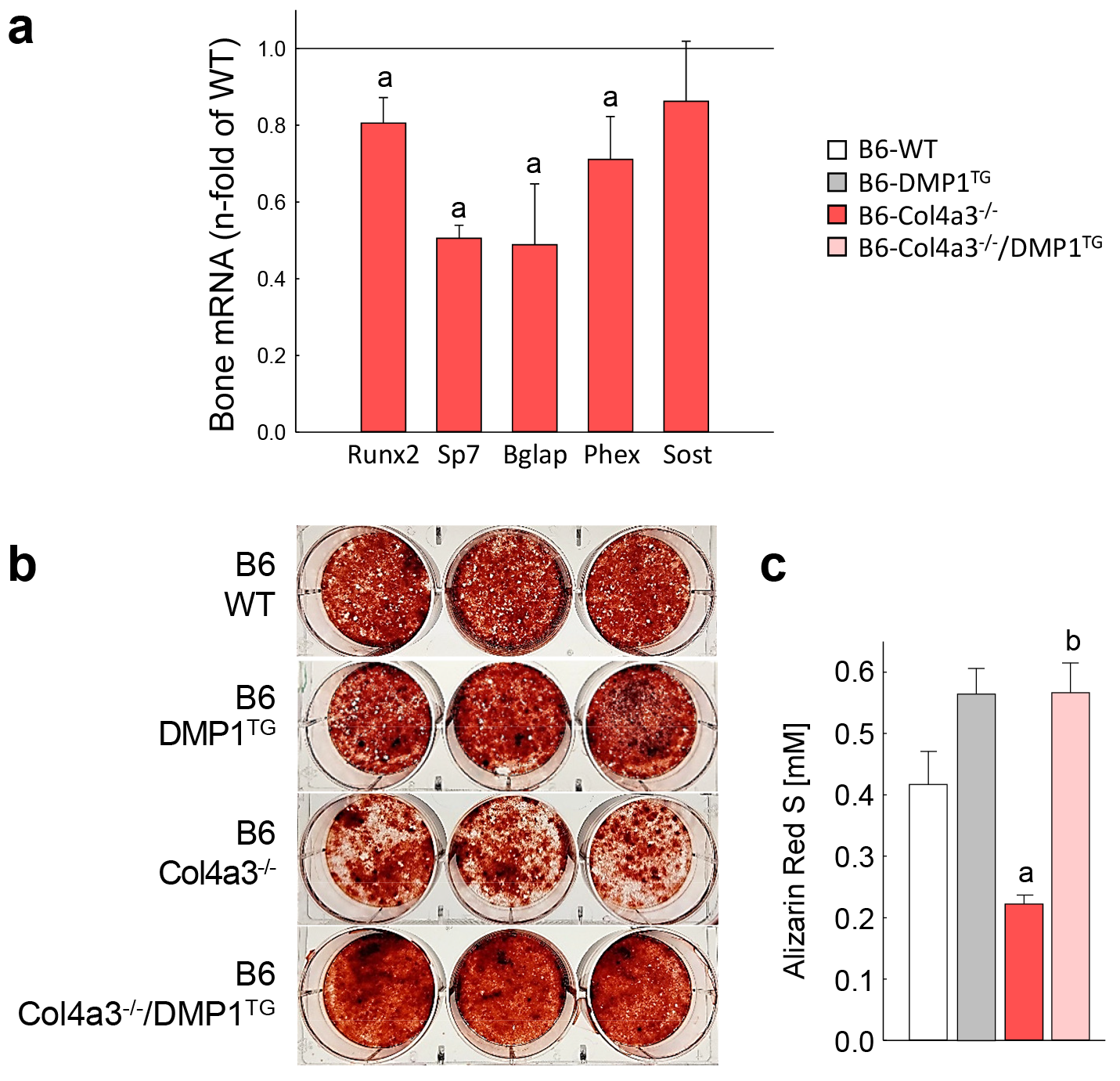

Supplement: Supplementary file 2 — Figure S1 [file 41413_2019_51_MOESM2_ESM.tif]

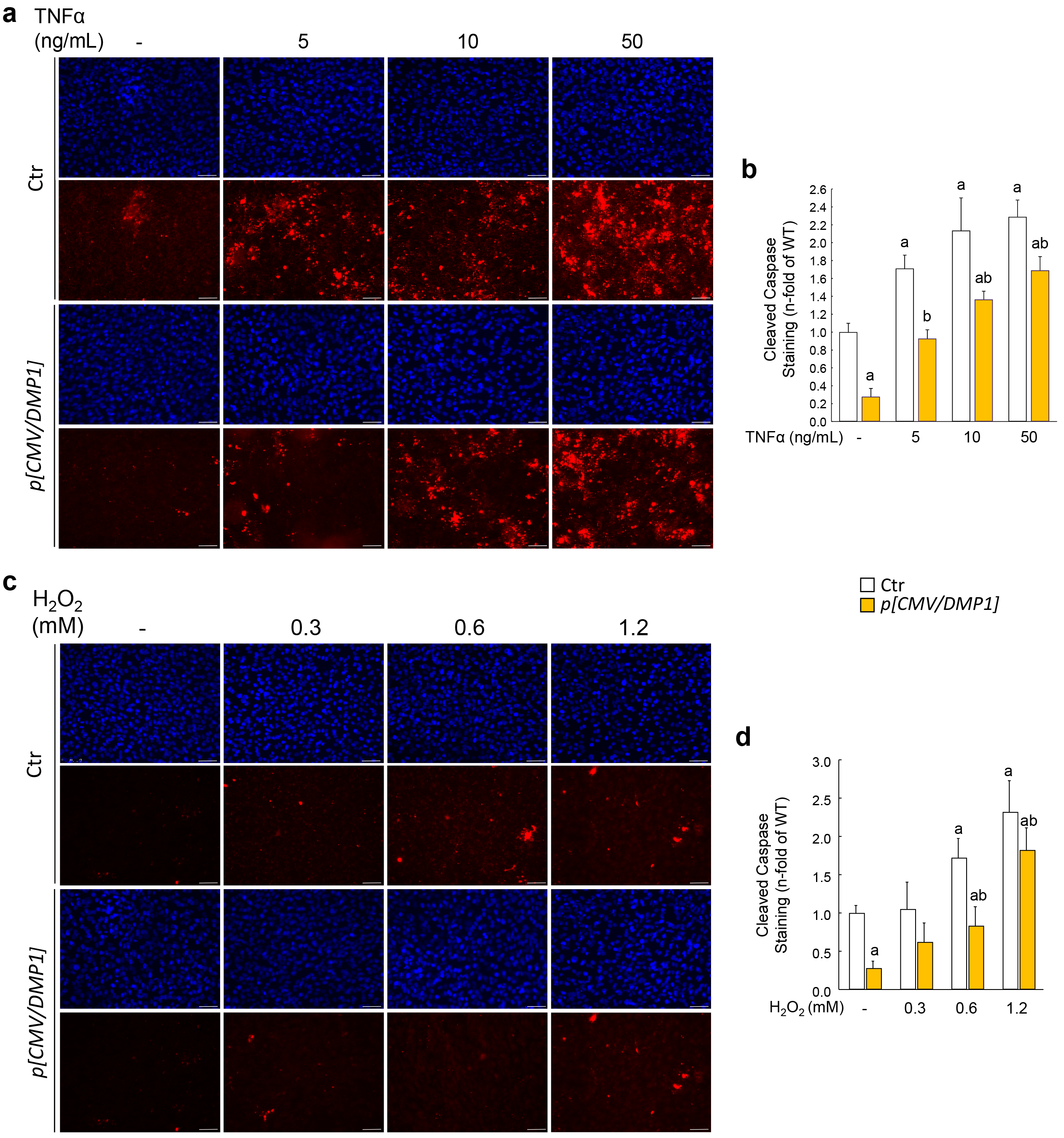

Supplement: Supplementary file 3 — Figure S2 [file 41413_2019_51_MOESM3_ESM.tif]

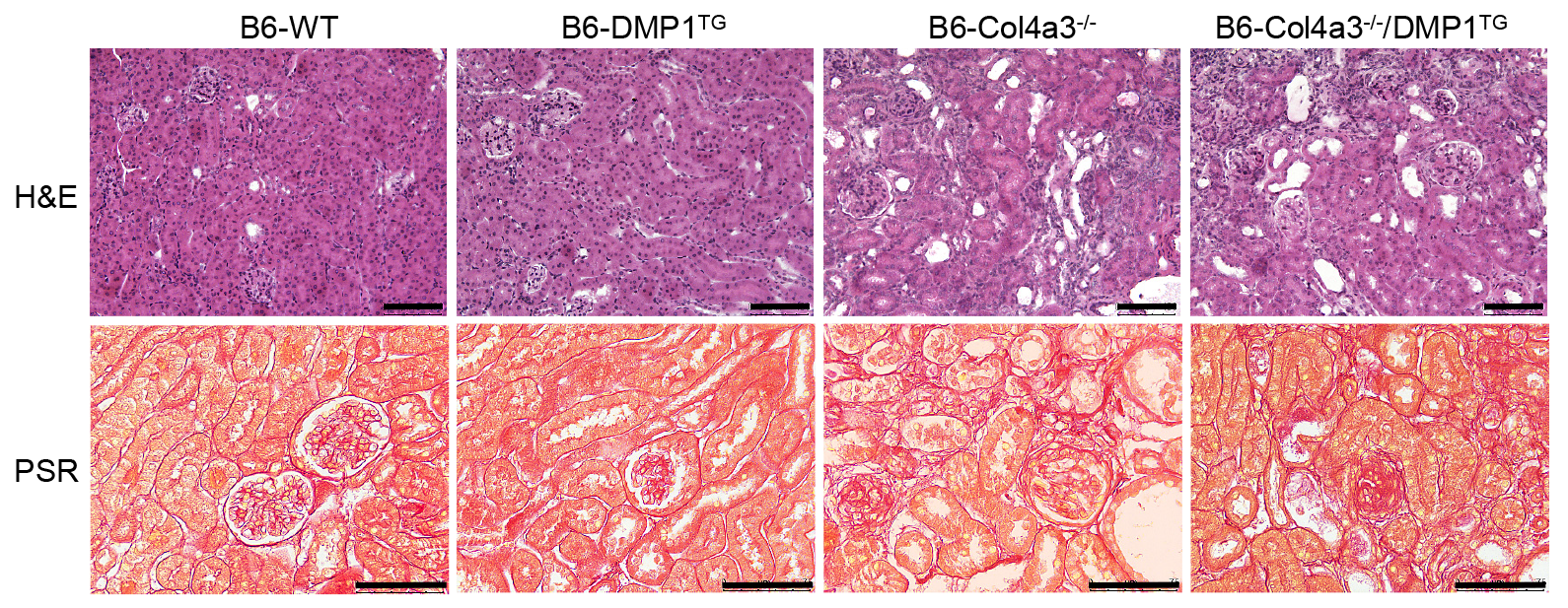

Supplement: Supplementary file 4 — Figure S3 [file 41413_2019_51_MOESM4_ESM.tif]

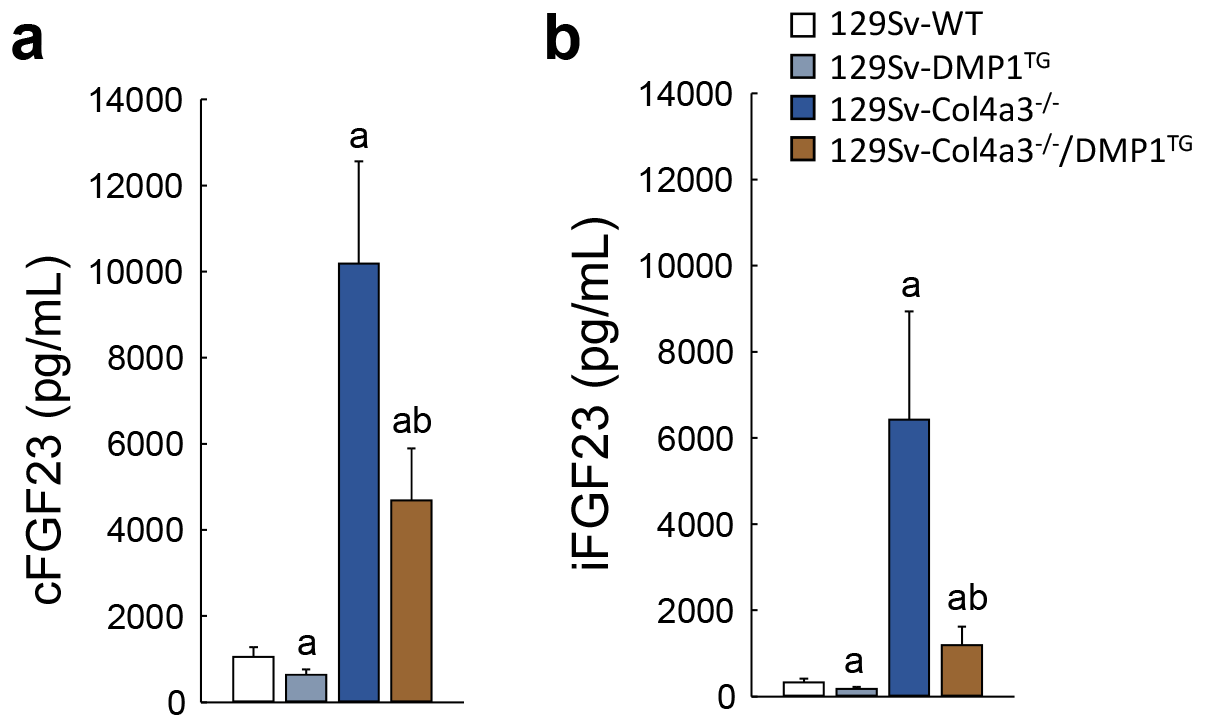

Supplement: Supplementary file 5 — Figure S4 [file 41413_2019_51_MOESM5_ESM.tif]

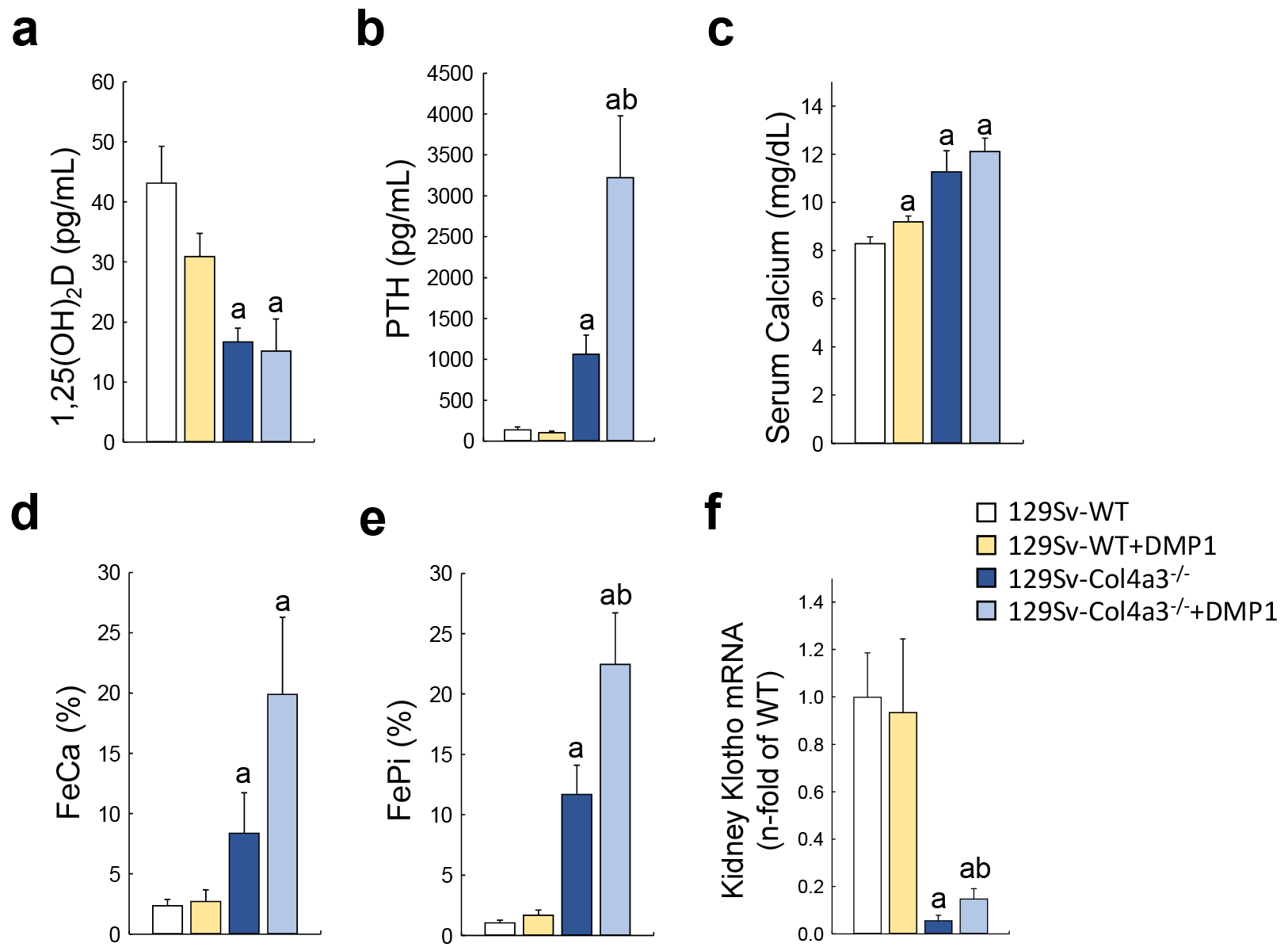

Supplement: Supplementary file 6 — Figure S5 [file 41413_2019_51_MOESM6_ESM.tif]
